# Relationships between family physicians’ referral for palliative radiotherapy, knowledge of indications for radiotherapy, and prior training: a survey of rural and urban family physicians

**DOI:** 10.1186/1748-717X-7-73

**Published:** 2012-05-18

**Authors:** Robert A Olson, Sonca Lengoc, Scott Tyldesley, John French, Colleen McGahan, Jenny Soo

**Affiliations:** 1 Department of Radiation Therapy, BC Cancer Agency, Vancouver Centre, Vancouver, Canada; 2 Department of Radiation Therapy, BC Cancer Agency, Centre for the North, Prince George, Canada; 3 Department of Surgery, Division of Radiation Oncology and Developmental Radiotherapeutics, University of British Columbia, Vancouver, Canada

**Keywords:** Cancer, Palliative radiotherapy, Family physician, Education, Knowledge, Palliative care

## Abstract

**Background:**

The primary objective of this research was to assess the relationship between FPs’ knowledge of palliative radiotherapy (RT) and referral for palliative RT.

**Methods:**

1001 surveys were sent to FPs who work in urban, suburban, and rural practices. Respondents were tested on their knowledge of palliative radiotherapy effectiveness and asked to report their self-assessed knowledge.

**Results:**

The response rate was 33%. FPs mean score testing their knowledge of palliative radiotherapy effectiveness was 68% (SD = 26%). The majority of FPs correctly identified that painful bone metastases (91%), airway obstruction (77%), painful local disease (85%), brain metastases (76%) and spinal cord compression (79%) can be effectively treated with RT, though few were aware that hemoptysis (42%) and hematuria (31%) can be effectively treated. There was a linear relationship between increasing involvement in palliative care and both self-assessed (p < 0.001) and tested (p = 0.02) knowledge. FPs had higher mean knowledge scores if they received post-MD training in palliative care (12% higher; p < 0.001) or radiotherapy (15% higher; p = 0.002). There was a strong relationship between FPs referral for palliative radiotherapy and both self-assessed knowledge (p < 0.001) and tested knowledge (p = 0.01).

**Conclusions:**

Self-assessed and tested knowledge of palliative RT is positively associated with referral for palliative RT. Since palliative RT is underutilized, further research is needed to assess whether family physician educational interventions improve palliative RT referrals. The current study suggests that studies could target family physicians already in practice, with educational interventions focusing on hemostatic and other less commonly known indications for palliative RT.

## Background

Radiotherapy (RT) is an effective palliative treatment in many scenarios faced by patients with metastatic cancer, including pain from local or metastatic disease, bleeding, airway obstruction, brain metastases, superior vena cava obstruction, and spinal cord compression [1-5]. RT in these scenarios can improve quality of life, and in some circumstances improve survival outcomes [4,6,7]. However, palliative RT is often underutilized, potentially because of a lack of knowledge of the indications for it.

Family physicians (FPs) are the primary caregivers of patients with metastatic cancer in many jurisdictions, including most of Canada, and are therefore the physicians in the best position to identify patients who may benefit from palliative RT and initiate a referral [8,9]. Previous research has shown that FP knowledge about the indications for palliative radiotherapy is limited and varied [10-12]. A review of the literature suggests that research in radiation oncology education is limited [13]. Research has also shown that RT is underutilized in many areas, and is most pronounced in areas remote from a cancer centre [8,13-15]. A relationship between underutilization of palliative radiotherapy and limited exposure to radiation oncology has been suggested; however, the emphasis in the literature has been on medical school exposure, with relatively limited research assessing post-MD exposure to radiation oncology or palliative care [11-13].

British Columbia (BC) is the third largest province in Canada with an area of 950,000 km^2^, and a population of 4.4 million. In 2010, there were over 21,000 new cases of cancer in British Columbia, and it is estimated that 60% of cancer diagnoses will ultimately meet indications for palliative RT [16,17]. There is a split between urban areas (with census areas of > 100,000 population) in the southwest and a mixture of suburban communities (with census areas of 10,000 to 100,000 population) and remote rural areas (census areas < 10,000 population) as described elsewhere [14]. The BC Cancer Agency (BCCA) is the sole provider of RT in BC, which is universally publicly funded, and delivered through one of five cancer centres, all of which are currently situated in southern urban centres (Vancouver, Victoria, Surrey, Kelowna, and Abbotsford).

The primary purpose of this research was to assess the relationships between FPs’ referral for palliative RT, knowledge of indications for palliative RT, and prior training in palliative care and radiotherapy. We hypothesize that knowledge of indications for radiotherapy is positively correlated with referral for radiotherapy and prior training. Secondary objectives were to assess the relationships between respondent characteristics tested knowledge of RT indications, and self-assessed knowledge of RT indications.

## Methods and materials

A survey previously described in the literature was adapted for BC physicians, after permission from the author, and mailed to all FPs from Northern BC and a random sample from greater Vancouver [11]. Respondents were provided with a stamped return envelope. The adapted survey is attached as an additional file 1. The primary purpose of distributing the adapted survey was to compare referral rates for radiotherapy from Northern BC FPs (rural) versus Greater Vancouver FPs (urban), since previous research by the group suggested referral rates are lower from rural BC [9]. The rural versus urban comparison has been presented elsewhere [15]. The survey also assessed FPs knowledge of radiotherapy indications, and previous training, which forms the basis of this analysis, which is to assess the relationships between knowledge of radiotherapy indications, referral for radiotherapy, and previous training. The emphasis is not on urban versus rural family physicians, though the inclusion of both potentially improves the generalizability.

A “tested knowledge score” is used for analysis, and is simply the count of number of correct answers from the seven questions asking FPs to rate the effectives of radiotherapy in several scenarios: “don’t know” or “not effective” were considered incorrect answers for each scenario, while either “somewhat effective” or “very effective” were considered equally correct answers. The maximum possible score was thus 7/7, and scores are presented as percentages. Respondents were also asked to rate their self-assessed knowledge of radiotherapy indications (1 = very little knowledge; 2-4 = somewhat, moderately, and extremely knowledgeable, respectively), which was summated to create a composite self-assessed knowledge score, with a maximum possible score of 12/12, which was converted to a percentage for figure creation.

FPs were identified through the college of physicians and surgeons of BC mailing list. Surveys were sent to all FPs in Northern BC (n = 351), which is predominantly rural and suburban, as well as a random sample of 650 FPs in greater Vancouver, which is predominantly urban and metropolitan (random sampling by Microsoft Excel. 2010). Surveys were re-sent one month later.

The total sample size was calculated for the primary purpose of the survey mail-out, and described above [15]. Of the fifty-four hundred FPs in BC, 351 practice in the Northern Health Authority and 1683 practice in the Vancouver Coastal Health Authority. Estimated sample sizes of 175 FPs in the North and 325 urban FPs ensure 90% power to detect a difference in proportion of individuals who refer for palliative radiotherapy at the 5% significance level, using estimated proportions of 40% and 55% for rural and urban physicians, respectively [18]. The test statistic used was a 2-sided normal approximation with pooled variance. Assuming a 50% survey response rate, a total of 1001 surveys were sent to FPs practising in British Columbia; 351 to northern FPs and 650 to urban FPs. 101 surveys were returned unopened because the respondents moved or retired, and were not included in the denominator calculating the response rate. Respondents who did not practice family medicine or where at least 80% of their practice was spent with either obstetrical or pediatric patients were asked not to complete the survey, and were excluded, as the primary purpose of the survey mail-out was to assess referral rates [15].

Descriptive statistics were used to present the knowledge scores. The correlation between the composite self-assessed knowledge score and the tested knowledge score was assessed with Pearson’s rank correlation coefficient. The relationship between mean knowledge scores and binary categorical variables were assessed with t-tests. Assessment for a linear relationship between knowledge scores and ordinal variables were assessed through ANOVA and eta squared. Multivariable analyses assessing the relationship between knowledge and self-reported referral for RT was performed using linear regression. All analyses were completed using SPSS Statistics GradPack 17.0 (Chicago, IL, USA).

This study was approved by the BCCA/UBC Research Ethics Board.

## Results

### Patient characteristics

There was a 33% overall response rate (n = 298); 55 respondents were excluded because they did not practice family medicine or at least 80% of their practice was spent with either obstetrical or pediatric patients, and as per survey instructions, they did not complete the questions. The median years in family practice was 21 (range 0 – 52). Table 1 summarizes the remainder of the respondent characteristics.

**Table 1:** Selected respondent characteristics

**Characteristic**		**Percentage of respondents**
Involved in palliative management of patients	Never	2%
	Rarely	18%
	Sometimes	35%
	Often	46%
Family practice in rural or suburban areas		50%
Aware of BCCA radiotherapy program		85%
Have ever referred a patient for radiotherapy		63%
Medical school training in	Palliative Care	44%
	Radiotherapy	18%
Post-MD training in	Palliative Care	28%
	Radiotherapy	6%

### Self-assessed and tested knowledge of palliative radiotherapy

**Figure 1:**
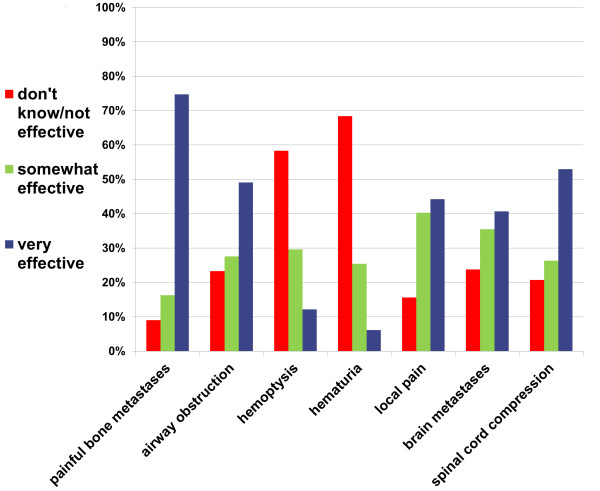
**Family physicians’ rating of effectives of radiotherapy in numerous scenarios**.

Figure 1 displays FPs opinions on the effectiveness of radiotherapy, and is the basis for the “tested knowledge score” as described above (percentage of correct answers, with “somewhat” or “very” effective considered equally correct). Respondents’ mean test score assessing their knowledge of palliative radiotherapy indications was 68% (SD = 26). The majority of FPs correctly identified that painful bone metastases (91%), airway obstruction (77%), painful local disease (85%), brain metastases (76%) and spinal cord compression (79%) can be effectively treated with RT, though few FPs were aware that hemoptysis (42%) and hematuria (31%) can be effectively treated with RT (Figure 1). Table 2 presents respondents’ self-assessed knowledge of palliative radiotherapy (second column) and forms the basis for a simple composite score of self-assessed knowledge (1–4 points for “very little”, “somewhat”, “moderately”, and “extremely” knowledgeable, respectively; summated to a maximum possible score of 12. Table 2 also demonstrates there is a highly significant linear relationship between self-assessed knowledge of RT and tested knowledge of palliative radiotherapy indications.

**Table 2:** Self-assessed knowledge of palliative radiotherapy and correlation with tested knowledge of palliative radiotherapy

**Self-perceived knowledge of:**		**Percentage of respondents**	**Mean tested knowledge score**	**P value for linear association**
Conditions for use of palliative RT	Very little	23%	52%	<0.001
	Somewhat	50%	72%	
	Moderately	27%	76%	
	Extremely	0%	-	
Benefits of palliative RT	Very little	12%	41%	<0.001
	Somewhat	46%	66%	
	Moderately	42%	77%	
	Extremely	<1%	85%	
Side effects of RT	Very little	12%	47%	<0.001
	Somewhat	50%	67%	
	Moderately	37%	76%	
	Extremely	1%	90%	

### Relationship between knowledge and referral for palliative radiotherapy

**Figure 2:**
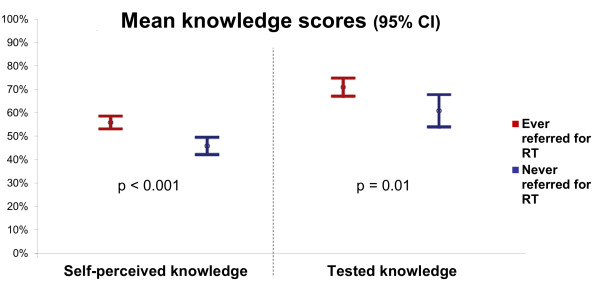
**Relationship between family physicians’ mean knowledge scores and self-reported referral for palliative radiotherapy (RT)**.

FPs who have ever referred for palliative radiotherapy have higher mean self-assessed knowledge scores (6.8 vs. 5.8 out of 12; p < 0.001; Figure 2). Likewise, FPs who have ever referred for palliative RT have higher mean tested knowledge scores (72% vs. 62%; p = 0.01; Figure 2). After controlling for geography of practice (rural/suburban Northern BC versus urban/metropolitan Greater Vancouver) and years of practice, both higher self-assessed knowledge score (odds ratio [OR] = 1.28 per unit increase; 95% confidence interval [CI] = 1.11 – 1.47; p = 0.001) and higher tested knowledge scores (OR = 1.01 per percentage increase; 95% CI = 1.00 – 1.02; p = 0.03) were positively associated with having ever referred for palliative RT.

### Relationship between knowledge and respondent characteristics

**Figure 3:**
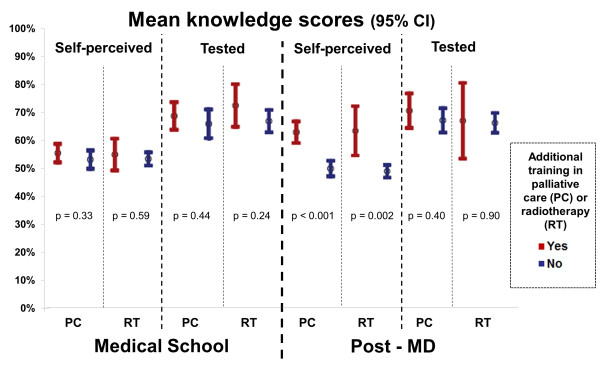
**Mean knowledge scores of family physicians depending on whether or not the received additional training in palliative care or radiotherapy during medical school or post-MD as a practicing physician**.

The mean number of years in FP was weakly positively correlated with the mean tested knowledge of palliative radiotherapy score (r = 0.16; p = 0.02). There was no significant relationship between geography of residence (urban/metropolitan Greater Vancouver vs. rural/suburban Northern BC) and mean self-assessed knowledge scores (6.4 vs. 6.4 out of 12; p = 0.95) or mean tested knowledge of palliative radiotherapy scores (67% vs. 69%; p = 0.46). There was a positive linear relationship between degree of involvement in palliative care and tested knowledge scores (Table 3), as well as self-assessed knowledge scores (p < 0.001). As a general trend, additional training in palliative care or radiotherapy is associated with higher mean knowledge scores (Figure 3). There were no significant relationships found between FPs knowledge and medical school training in palliative care or radiotherapy (Figure 3). Likewise, there were no statistically significant relationships between mean tested knowledge scores and additional training in the post-MD setting (Figure 3). Respondents had significantly better self-assessed knowledge scores if they reported post-MD training in palliative care (7.5 vs. 6.0 out of 12; p < 0.001) or radiotherapy (8.1 vs. 6.3 out of 12; p = 0.002).

**Table 3:** Relationship between tested knowledge of palliative radiotherapy and involvement in palliative care of patients

**Involvement in palliative care**	**Mean tested knowledge scores**	**P value for linear association**
Never	48%	0.02
Rarely	61%	
Sometimes	68%	
Often	71%	

## Discussion

This survey of FPs from rural, suburban, and urban BC demonstrates that their tested knowledge of palliative radiotherapy indications is correlated with their self-assessed knowledge of palliative radiotherapy. It also demonstrated that FPs undervalue the role of palliative RT beyond the obvious circumstance of painful bone metastases, mirroring the knowledge gap found in US palliative care physicians[19]. Scores on tests of indications for palliative RT, and self-assessed knowledge of palliative RT, are higher in those with prior training in radiotherapy or palliative care. Furthermore, knowledge (both self-assessed and tested) of palliative radiotherapy is highly correlated with referral for RT. Though the retrospective nature of this study cannot assess causality, these results support further research assessing whether improved FP education in palliative RT will increase utilization of this treatment, with a potential to improve the quality of life of patients with metastatic cancer.

There are several potential explanations for the relationship seen between knowledge of palliative RT and referral for RT. The most plausible explanation is that formal education in palliative RT indications results in improved knowledge of indications for palliative RT, and in turn results in increased referral for RT. Second, the causality may be reversed; FPs who have encountered patients with metastatic cancer and referred for palliative RT have improved knowledge of RT indications as result, potentially through reading motivated by the patient encounter or education received from radiation oncologist consultation letters. Third, there may not be a causal relationship between knowledge and referral for RT, but rather both knowledge and referral may be correlated to an unmeasured factor, such as patient volume. Likely, the relationship identified in this study is a combination of all of these explanations, though the nature of this study design does not allow for assessment of causality.

Given the relationship seen between FP knowledge of palliative RT and referral for palliative RT in both this and other studies, future research should assess whether educational interventions aimed at FPs improve referral and utilization of palliative RT. Given the high scores on the analgesic compared to haemostatic properties of RT observed, it would potentially be more beneficial to focus on improvement in education of less well known palliative RT indications such as bleeding, rather than commonly known indications such as painful bone metastases. However, given that the response rate to our survey is only 33%, we are unable to assess the knowledge of the remaining 67% of physicians, whose knowledge may have an impact on the overall rate of referral for palliative RT. Certainly, education on common indications for palliative RT is important and necessary, and therefore should not be omitted [13]. Furthermore, these results suggest that FPs in practice, rather than medical students, may be the more appropriate target audience for future studies.

There are several potential methods to improve education and potentially the utilization of palliative RT. Conventional methods aimed at medical students or FPs through continuing medical education sessions, whether virtual or in-person have been assessed in multiple medical fields [13]. Several authors have reported on education of medical students, showing short term improvements in knowledge, with limited evidence to date that this will translate into long term practice changes for those who choose FP [20-23]. Dennis and Duncan have recommended that educational interventions for medical students should focus on general knowledge that is applicable to future FPs, rather than detailed information more relevant to oncologists [13]. More novel techniques to both educate and improve appropriate referral to specialists have also been developed. For example, in BC a “shared care” initiative is currently under investigation where both FP and specialists are compensated for telephone advice on the appropriate management and referral of patients [24,25].

The results of this research should be considered in the context of its strengths and limitations. The reasonable response rate and equal proportions of FPs from rural/suburban versus urban/metropolitan areas improves the generalizability of the results. Given the BCCA was the sole provider of RT, in a jurisdiction with universally publicly funded health care and salaried radiation oncologists, referrals are not influenced by competition between RT providers or compensation of FPs or radiation oncologists. The assessment of knowledge of palliative RT indications is limited by the fact that the investigators interpreted respondents’ answer of “somewhat” or “very effective” as a respondent’s belief that RT was indicated in that scenario, since indication for RT was not directly asked. Also, the nature of the survey study design limits several interpretations of the results. First, causality between knowledge and referral for palliative RT cannot be assessed. Second, survey research is prone to response bias, where respondents with certain characteristics (such as interest in oncology) may be more likely to return surveys, and thereby result in higher mean tested and self-assessed knowledge scores than may exist in the true population [26]. Third, recall bias is common in survey research, and for this study theoretically could result in an under-reporting of previous referral for RT [27]. However, many of these limitations are common to all survey research, and the effort placed to sample FPs from rural through metropolitan practices broadens the generalizability in comparison to most survey research which focuses on academic physicians.

## Conclusion

Family physician self-assessed and tested knowledge of palliative radiotherapy and its indications is positively associated with increased referral for palliative radiotherapy. Since palliative radiotherapy is underutilized, further research is needed to assess whether family physician educational interventions improve palliative radiotherapy referrals. The current study suggests that education regarding less common palliative radiotherapy indications to family physicians already in practice is a reasonable place to start this research.

## Competing interests

The author(s) declare that they have no competing interests.

## Authors’ contributions

All authors were involved in the study design and implementation methodology. RO conceived of and led the analysis, and drafted the manuscript. CM provided statistical support. All authors read, provided intellectual editing, and approved the manuscript.

## Authors’ information

RO and ST are both clinician scientist radiation oncologists at the BC Cancer Agency. CM is the biostatistical lead of the cancer surveillance & outcomes division of the BC Cancer Agency. JF is the director of clinical operations for the radiotherapy program at the BC Cancer Agency. JS and SL are radiation therapists at the Vancouver Cancer Centre.

## Supplementary Material

Additional file 1 Survey Questions asked of Family Physicians. Click here for file
