# Supplementary material for: Relationships between family physicians’ referral for palliative radiotherapy, knowledge of indications for radiotherapy, and prior training: a survey of rural and urban family physicians
Source: Radiat Oncol. 2012 May 18;7:73. doi: 10.1186/1748-717X-7-73 (PMC3484047; doi:10.1186/1748-717X-7-73)
Supplement: Additional file 1 — Survey Questions asked of Family Physicians. [file 1748-717X-7-73-S1.doc]

**Additional File: Survey Questions asked of Family Physicians**

*If you are not currently practicing family medicine or do not have any patients with cancer in your practice, please check the appropriate box.*

**** I do not practice family medicine.

**** 80% of my practice is spent with either obstetrical or pediatric patients.

*If you checked any of the above two boxes, you do not need to complete the rest of the form,* ***but*** *please return the survey in the envelope provided*.

1. **British Columbia Cancer Agency (BCCA) Awareness**

Are you aware of the BCCA’s Radiation Oncology Program?

**** Yes **** No

Have you ever obtained advice from the radiation oncologists at BCCA?

**** Yes **** No

If **yes**, what kind of advice? *Please check all that apply.*

- - - How to make a referral to the BCCA
    - To determine suitability of a referral
    - To discuss unanticipated side effects of treatment
    - To discuss current plan of management
    - Other, please specify:

1. **Radiation Therapy**

Have you encountered a patient with cancer in your practice who would or could have benefited from palliative radiation treatment in the past year?

**** Yes **** No

Have you ever referred a patient for radiation therapy at BCCA?

**** Yes **** No

How would you rate the effectiveness of radiation therapy for the treatment of the following cancer related symptoms? *Please* ***circle*** *the most appropriate response.*

|  | **Not****Effective** | **Somewhat**  **Effective** | **Very**  **Effective** | **Don’t**  **Know** |
| --- | --- | --- | --- | --- |
| a. Painful bony metastases | 1 | 2 | 3 | 4 |
| b. Airway obstruction due to tumor | 1 | 2 | 3 | 4 |
| c. Hemoptysis | 1 | 2 | 3 | 4 |
| d. Hematuria | 1 | 2 | 3 | 4 |
| e. Painful local disease (e.g. pelvis mass) | 1 | 2 | 3 | 4 |
| f. Brain metastases | 1 | 2 | 3 | 4 |
| g. Spinal cord compression | 1 | 2 | 3 | 4 |

Please rate your knowledge of each of the following. *Please* ***circle*** *the most appropriate response.*

|  | **Very Little Knowledge** | **Somewhat Knowledgeable** | **Moderately Knowledgeable** | **Extremely Knowledgeable** |
| --- | --- | --- | --- | --- |
| 1. Conditions for which there is evidence that radiation therapy can be used as part of curative cancer treatment | 1 | 2 | 3 | 4 |
| 1. Potential benefits of palliative radiotherapy | 1 | 2 | 3 | 4 |
| 1. Potential side effects associated with radiation therapy | 1 | 2 | 3 | 4 |
| 1. Management of common radiotherapy side effects | 1 | 2 | 3 | 4 |

1. **Respondent Profile**

During your medical training, did you receive any formal training (e.g., lectures, mentorships or traineeships) in palliative care?

**** Yes **** No If **yes**, please indicate the number of weeks: ______

Did you receive any formal training in radiation oncology during your medical training?

**** Yes **** No If **yes**, please indicate the number of weeks: ______

Did you receive additional training in the following areas? *Please check all that apply.*

**** Palliative care **** Radiation therapy
